# Cellulitis in older people over 75 years – are there differences?

**DOI:** 10.1016/j.amsu.2019.11.012

**Published:** 2019-11-22

**Authors:** Manoj Kumar, Vincent Jiu Jong Ngian, Clarence Yeong, Caitlin Keighley, Huong Van Nguyen, Bin Soo Ong

**Affiliations:** a Bankstown-Lidcombe Hospital, Bankstown, New South Wales, Sydney, Australia; b University of New South Wales, Sydney, New South Wales, Australia

**Keywords:** Cellulitis, Older people, Outcomes

## Abstract

**Aim:**

To examine differences in risk factors, clinical features and outcomes of cellulitis between those 75 + years and those <75 years admitted to a metropolitan hospital.

**Methods:**

A prospective study of patients with limb cellulitis requiring intravenous antibiotics conducted at Bankstown-Lidcombe Hospital, Australia from June 2014 to April 2015.

**Results:**

Thirty one patients were 75 + years and 69 less than 75 years. A greater proportion of older patients resided in nursing home (25.8% vs 2.9% respectively, p = 0.001) and mobilized with walking aid(s) (58.1% vs 11.6% respectively, p < 0.001). Significantly more older patients had documented hypertension (45.2% vs 23.2% respectively p = 0.035), atrial fibrillation (33.5% vs 5.8% respectively, p < 0.001), dementia (22.6% vs 1.4% respectively, p = 0.001) and malignancy (16.1% vs 1.4% respectively, p = 0.010). The clinical presentation of cellulitis and cellulitis severity (Eron classification) did not significantly differ in both groups; however older patients were more likely to have dependent edema (OR 4.0, 95%CI 1.3–12.6, p = 0.018) and less likely to be obese (OR 0.3, 95%CI 0.1–0.8, p = 0.012) or had a past history of cellulitis (OR 0.3, 95%CI 0.1–1.0, p = 0.044) on presentation. Despite the age difference, there were no major differences in intravenous antibiotic choice, hospital length of stay, and hospital readmission rates in both groups. Older patients however, were more likely to experience complications such as falls and/or decreased mobility (38.7% vs 15.9% respectively, p = 0.020) during the cellulitis episode.

**Conclusion:**

There are minor differences in the risk factors and clinical features of cellulitis in older patients as compared to the young. Outcomes are similar except for a higher incidence of hospital related complications.

## Highlights

• To examine the differences in outcomes of cellulitis in hospitalized patients who are 75 + years and those <75 years.
• A prospective study of patients with limb cellulitis requiring intravenous antibiotics conducted at Bankstown Hospital.
• Older people had similar outcomes to younger patients after presenting to hospital with mild to moderate limb cellulitis.

## 1 Introduction

Cellulitis is a bacterial infection of the skin involving the dermis and subcutaneous fat. In Australia, cellulitis accounts for over 250,000 hospital bed days, or 10.5% of potentially preventable hospitalizations [1].While most episodes of cellulitis can be managed as an outpatient, a significant proportion, particularly older people, require hospitalization. Over a 12-month period from 2014 to 2015, the cellulitis hospitalization rate was 1100 per 100,000 in the 80 plus age group as opposed to 237 episodes per 100,000 in the general population [1].

Cellulitis typically presents with pain, erythema, warmth and edema. Systemic symptoms including fever and tachycardia may be present although thought to be less frequent in older persons [[2], [3], [4], [5], [6]]. Known risk factors for cellulitis are venous edema, lymphedema, skin conditions, traumatic injury, leg ulcers, peripheral vascular disease, fungal infections, past history of cellulitis and obesity [[7], [8], [9], [10]].

Age alone does not alter treatment principles for bacterial cellulitis (including use of antibiotics); however age-related pharmacokinetics and pharmacodynamics, cognitive status and social circumstances [11] may impact on treatment decisions particularly need for hospitalization.

Once hospitalized, age is an independent risk factor for increased length of stay for cellulitis with other factors being long duration of symptoms, tachycardia, hypotension, leukocytosis, hypoalbuminemia, elevated serum creatinine, bacteremia, obesity and diabetes mellitus [[12], [13], [14], [15]].

Age is significantly associated with increased mortality from cellulitis although it is unclear if this is due to illness severity or underlying comorbidity [16]. Other factors associated with mortality are delayed administration of antibiotics, presence of multiple comorbidities, previous myocardial infarction, congestive heart failure, liver disease, hypoalbuminemia, renal insufficiency, morbid obesity, lower limb edema, *Pseudomonas aeruginosa* infection, bacteremia and septic shock [14,17].

Hospital readmission for cellulitis is also more common in older people [18] particularly if there has been more than one prior episode of cellulitis^19.^

## 2 Aims

In this prospective study, we aimed to examine differences in risk factors, clinical features, management, and outcomes of cellulitis between those 75 years or more and those less than 75 years admitted to a large metropolitan hospital.

## 3 Methods

The study was conducted at Bankstown-Lidcombe Hospital, New South Wales, Australia from June 2014 to April 2015. The study was approved by the South-Western Sydney Local Health District (SWSLHD) Ethics Committee.

Between June 2014 and April 2015, potential patients were identified through review of the Bankstown Hospital inpatient list three times a week by a study investigator. We included all identified patients aged 18 years or more with a diagnosis of cellulitis of the upper and/or lower limb(s) and excluded patients with infected ulcers on presentation, pregnant patients and those with post-operative wound infections.

The patients were then stratified into an older group (aged 75 years or more) and a younger group (74 years or less) and were followed up during their admission and for a total of 28 days post completion of intravenous antibiotics. We studied the over 75 years age group as that this age group is more descriptive of the frail older cohort [19].

Data collected included basic demographics, clinical characteristics, relevant investigations, treatment provided and clinical outcomes. The severity of cellulitis was rated using the Eron classification [20].

Data were analyzed with SPSS Version 24 and R version 3.3.1. Chi-square test was used to compare proportions. Student's T-test was used to compare differences in means for normally distributed variables. For non-normally distributed continuous variables, non-parametric test was used to assess differences in the ranked median scores. Logistic regression was used to assess statistically significant risk factors for cellulitis in the older and younger age groups. Statistically significant results were set at an alpha level of 0.05. The study is in line with the STROCSS guidelines [21]. The study also been registered on the research registry UIN:researchregistry5125.

## 4 Results

One hundred and thirteen patients were identified during the study period and 100 patients (88.5%) consented to participate. Thirty-one (31.0%) patients were aged 75 years and older and 69 (69.0%) patients were 74 years or less.

The mean age was 84.4 ± 5.8 years in the older group and 53.4 ± 14.2 years in the younger group. The older patients had lower BMI than their younger counterparts [28.3 (±8.0) vs 36.0 (±12.3) respectively, p < 0.001]. A higher proportion resided in residential aged care facilities (25.8% vs 2.9% respectively, p = 0.001); and mobilized with walking aid(s) (58.1% vs 11.6% respectively, p < 0.001). (Table 1).

**Table 1:** Patient characteristics.

	Age <75 years (N = 69)	Age 75 + years (N = 31)	P-value
Age mean(SD)	53.4 (±14.2)	84.4 (±5.8)	<0.001
Female n (%)	23 (33.3%)	16 (48.5%)	0.12
BMI	36.0 (±12.3)	28.3 (±8.0)	<0.001
Residential Aged Care Facility	2 (2.9%)	8 (25.8%)	0.001
Mobility			<0.001
Mobile unaided	61 (88.4%)	13 41.9%)	
Mobile with aid	8 (11.6%)	18 (58.1%)	
**Risk factors**^a^ OR (95% CI)			
Dependent edema	–	4.0 (1.3–12.6)	0.018
Obesity (BMI>30)	–	0.3 (0.1–0.8)	0.012
Previous cellulitis	–	0.3 (0.1–1.0)	0.044
Peripheral vascular disease	–	3.1 (0.9–10.6)	0.079
Tinea pedis	–	0.9 (0.2–4.1)	0.930
Venous dermatitis	–	0.5 (0.1–2.0)	0.302
**Comorbidities**			
Hypertension	16 (23.2%)	14 (45.2%)	0.035
AF	4 (5.8%)	11 (33.5%)	<0.001
Dementia	1 (1.4%)	7 (22.6%)	0.001
Malignancy	1 (1.4%)	5 (16.1%)	0.010
Diabetes	23 (31.9%)	6 (19.4%)	0.24
IHD	11 (15.9%)	10 (32.3%)	0.11
CCF	10 (14.5%)	9 (29.0%)	0.10
DVT	3 (4.3%)	5 (16.1%)	0.10
PE	2 (2.9%)	5 (16.1%)	0.072
Steroid use last 3 months	3 (4.3%)	3 (9.7%)	0.37

a Logistic regression – Chi-square = 17.868, p = 0.007, df = 6, Nagelkerke's R2 0.230; BMI body mass index; IHD ischaemic heart disease; AF atrial fribrillation; CCF congestive cardiac failure; DVT deep vein thrombosis; PE pulmonary embolism.

A significantly higher proportion of older patients had documented hypertension (45.2% vs 23.2% respectively p = 0.035), atrial fibrillation (33.5% vs 5.8% respectively, p < 0.001), dementia (22.6% vs 1.4% respectively, p = 0.001) and malignancy (16.1% vs 1.4% respectively, p = 0.010). (Table 1).

In terms of cellulitis risk factors, after controlling for potential confounders, older patients were more likely to have dependent edema (OR 4.0 95%CI 1.3–12.6, p = 0.018); but less likely to be obese (OR 0.3, 95%CI 0.1–1.0, p = 0.012) or had a prior history of cellulitis (OR 0.3, 95%CI 0.1–1.0, p = 0.044) than younger patients. The risk of peripheral vascular disease, tinea pedis and cutaneous dermatitis were similar in both groups.

Cellulitis presenting features such as pain, fever, chills and vital signs (temperature, heart rate and blood pressure) did not significantly differ between the two groups. The severity of cellulitis, as defined by the Eron classification [22] also did not differ between groups with the majority of patients having Eron Classes I and II (Table 2**).**

**Table 2:** Clinical characteristics.

	Age <75 years (N = 69)	Age 75 + years (N = 31)	P-value
Duration of symptoms median (IQR)	3.0 (2.0–6.0)	4.0 (2.0–14.0)	0.23
Fever and chills	19 (27.5%)	6 (19.4%)	0.46
Heart rate	92 (±17)	89 (±15)	0.384
BP – Systolic	136 (±18)	143 (±24)	0.158
BP _Diastolic	75 (±12)	72 (±11)	0.227
Temperature	37.4 (±1.0)	37.3 (±1.0)	0.796
**Pain score**			0.055
Mild 0-3	32 (51.6%)	14 (53.8%)	
Moderate 4-7	20 (32.3%)	12 (46.2%)	
Severe 8-10	10 (16.1%)	0	
**Pathology**			
Haemoglobin	135.0 (±19.4)	122.1 (±16.4)	0.002
White cell count	11.3 (±5.1)	11.1 (±6.0)	0.61
Albumin	41.4 (±4.1)	38.0 (±4.7)	0.001
Creatinine – median (IQR)	88 (76–106)	93 (71–131)	0.692
Urea – median (IQR)	5.8 (4.8–8.4)	7.9 (5.8–12.4)	0.011
CRP – median (IQR)	33 (15–117)	60 (9–133)	0.919
Blood culture	29 (42.0%)	18 (58.1%)	0.071
**Eron Classification**			0.415
Class I	10 (14.5%)	2 (6.5%)	
Class II	55 (79.7%)	26 (83.9%)	
Class III	4 (5.8%)	3 (9.7%)	
Class IV	0	0	

BP blood pressure; CRP C reactive protein.

Initial laboratory results revealed that older patients had lower hemoglobin [122.1 (±16.4) vs 135.0 (±19.4), p = 0.002] and albumin [38.0 (±47) vs 41.4 (±4.1), p < 0.001] and higher urea level [7.9 (5.8–12.4) vs 5.8 (4.8–8.4), p = 0.011] compared to their younger counterparts. CRP white cell count (WCC) and positive rate of blood culture did not differ between the two groups (Table 2**).**

Older inpatients presenting with cellulitis were less likely to be referred to hospital in the home (HITH) antibiotic programs for completion of the course of intravenous antibiotics 32.3% vs 59.4% respectively, p = 0.012) compared to younger patients. The antibiotic choices did not differ between the two populations, these included Cephazolin, Flucloxacillin or Tazobactam-Piperacillin.

Older patients with cellulitis were more likely to experience falls or decreased mobility (38.7% vs 15.9% respectively, p = 0.020) compared to the younger group. (Table 3). Despite this, they had similar LOS to their younger counterparts [10 (7–15) vs 8 (6–13) respectively, p = 0.403]. There was one death in each group and the rates of ICU admission, surgical intervention and 28-day readmission were similar in the two groups.

**Table 3:** Treatment, complications and outcomes.

Characteristics	Age <75 years (N = 69)	Age 75 + years (N = 31)	P-value
Completed treatment via HiTH	41 (59.4%)	10 (32.3%)	0.012
Duration of IV antibiotic – median (IQR)	6 (4–8)	4 (2–9)	0.059
Length of hospital stay – median (IQR)	8 (6–13)	10 (7–15)	0.403
Antibiotics			0.121
Cephazolin	47 (51.1%)	16 (32.7%)	
Flucloxacillin	20 (21.7%)	12 (24.5%)	
Tazobactam-piperacillin	7 (7.6%)	4 (8.2%)	
Complications			
DVT	0	0	1
PE	0	1 (3%)	0.31
Fall or decreased mobility	11 (15.9%)	12 (38.7%)	0.020
Nosocomial infection	1 (1.4%)	3 (9.7%)	0.087
Delirium	1 (1.4%)	2 (6.5%)	0.23
Outcomes			
Death	1 (1.4%)	1 (3.0%)	0.531
Needing surgical intervention	3 (4.3%)	2 (6.5%)	0.644
ICU admission	0	0	1
Readmission within 28 days	4 (5.8%)	0	0.308
Duration of IV antibiotic – median (IQR)	6 (4–8)	4 (2–9)	0.059
Length of hospital stay – median (IQR)	8 (6–13)	10 (7–15)	0.403
Antibiotics			0.121
Cephazolin	47 (51.1%)	16 (32.7%)	
Flucloxacillin	20 (21.7%)	12 (24.5%)	

HiTH hospital in the home; DVT deep vein thrombosis; PE pulmonary embolism.

## 5 Discussion

In this study, we found that older people, despite being frailer than their younger counterparts, had similar treatment outcomes after presenting to hospital with mild to moderate limb cellulitis.

In our study, most of the potential risk factors for cellulitis were similar in the older and younger age groups; however, older patients were more likely to have dependent edema and impaired mobility, and less likely to be obese. Other conditions noted to be more common in the older group were congestive cardiac failure, atrial fibrillation, dementia and malignancy. We believe this finding reflected the higher prevalence of these conditions in the older population rather than an association with cellulitis.

Over 25% of older patients with cellulitis lived in residential aged care facilities. This finding raised the opportunity for the provision of ambulatory care antibiotic programs in aged care homes potentially avoiding the need for hospitalization for residents with cellulitis.

There were no significant differences in the clinical presentation of cellulitis between the two age groups (i.e., duration of cellulitis symptoms, heart rate, blood pressure, temperature, white cell count, CRP and Eron severity classification). Atypical and blunted physiological response to infection with age has been documented in the literature [23]. In severe sepsis, a reduced physiological response can lead to rapid progression of sepsis [2,3,22].Our results did not support a blunted response to infection in older patients with cellulitis. We, however, did not have any patients with severe sepsis to examine the inflammatory response in more detail.

In our study, older patients experienced more falls and impaired mobility during the admission for cellulitis compared to younger patients. While these factors might have made their hospital discharge planning more complex, they did not translate into an increased hospital length of stay. Previously described risk factors affecting LOS in cellulitis *(comprising of age, hypoalbuminemia, bacteremia, obesity, diabetes mellitus, tachycardia, hypotension, leukocytosis, and elevated serum creatinine)* [7,[12], [13], [14], [15]], tended to be skewed towards age and hypoalbuminemia for the older group and obesity for the younger group in our study.

There were no statistically significant differences between the two groups in terms of mortality, ICU admission, and surgical intervention for cellulitis complications. The majority of patients in both groups had Eron Class I or II cellulitis and did not sustain physiological decompensations; however, in more severe cases of cellulitis, one would expect ageing physiology to sustain more physiological decompensations which may then influence the above parameters.

A lower proportion of older inpatients discharged to HITH programs might have been attributable to their medical comorbidities and functional criteria not meeting HITH requirements. As such, additional health resources may allow HITH programs to manage these complex patients but this would require further study.

Unlike previous published literature [18], we did not find a significant difference in the 28-day readmission rate between the two age cohorts in our study. As the readmission rate was less than 5%, a study with greater number of patients would have more power to detect small differences in readmission rates.

One of the limitations of this study is the small sample size due to a short recruitment period; further study with a larger sample size would assist in validation of our findings. We decided to focus on inpatient cellulitis treatment; however a cellulitis management journey from hospital to community settings would have provided with a more complete picture.

As the number of older patients presenting with cellulitis increases as the population ages, it is important to note that for mild to moderate cellulitis, older patients perform just as well as younger patients with standard cellulitis treatments on clinical and care indicators.

We wish to confirm that there are no known conflicts of interest associated with this publication and there has been no financial or person support for this work with any other people or organizations.

## Ethical approval

Ethics approval was taken from South-Western Sydney Local Health District (SWSLHD) Ethics Committee.

## Sources of funding

No funding was obtained for the research.

## Author contribution

Bin Soo Ong – Supervisor, study concept.

Manoj Kumar – Writing the paper.

Huong Van Nguyen – writing the paper.

Clarence Yeong – Data collection.

Vincent Ngian – data analysis, study concept.

Caitlin Keighley – study concept, data collection.

## Research registration number

1. Name of the registry: Research Registry.

2. Unique Identifying number or registration ID: researchregistry5125.

3. Hyperlink to the registration (must be publicly accessible): https://www.researchregistry.com/browse-the-registry#home/registrationdetails/5d7a4d440ba5620010271dc1/

## Guarantor

Dr Manoj Kumar.

## Consent

All authors have consented to publication.

## Provenance and peer review

Not commissioned, externally peer reviewed.

## Declaration of competing interest

No conflict of interest from any of the authors.
